# Novel Kalman Filter Algorithm for Statistical Monitoring of Extensive Landscapes with Synoptic Sensor Data

**DOI:** 10.3390/s150923589

**Published:** 2015-09-17

**Authors:** Raymond L. Czaplewski

**Affiliations:** Emeritus Scientist, U.S. Forest Service, Rocky Mountain Research Station, Fort Collins, CO 80521, USA; E-Mail: raymond_czaplewski@environmetrika.org; Tel.: +1-970-218-5907

**Keywords:** Landsat, MODIS, change detection, square root filter, big data, forest inventory and analysis program, FIA

## Abstract

Wall-to-wall remotely sensed data are increasingly available to monitor landscape dynamics over large geographic areas. However, statistical monitoring programs that use post-stratification cannot fully utilize those sensor data. The Kalman filter (KF) is an alternative statistical estimator. I develop a new KF algorithm that is numerically robust with large numbers of study variables and auxiliary sensor variables. A National Forest Inventory (NFI) illustrates application within an official statistics program. Practical recommendations regarding remote sensing and statistical issues are offered. This algorithm has the potential to increase the value of synoptic sensor data for statistical monitoring of large geographic areas.

## 1. Introduction

An official statistics program, such as a National Forest Inventory (NFI), is a large and complex interdisciplinary enterprise. It involves three different but closely interconnected disciplinarians: the remote sensing technologist, the resource analyst, and the sample survey statistician. Each discipline has different objectives, perspectives, and technical issues.

The remote sensing technologist produces synoptic geospatial databases that characterize certain conditions of land cover and changes in that cover over time and space. Within an NFI, examples include a few characteristics like land cover, woody biomass, and forest stand conditions. The technologist builds statistical models that use synoptic sensor data to predict those forest attributes for each mapping unit, such as a pixel or polygon. The resource analyst may use those geospatial predictions to gain a better qualitative understanding of the spatial and temporal patterns of major disturbances and land cover changes in the target population. Furthermore, enumeration of predictions for every mapping unit in the study area produces census statistics, such as the proportion of the study area with forest cover, as predicted with sensor data. The statistician may use those census data as auxiliary information to improve accuracy and reduce uncertainty in estimated population statistics for the study area.

The resource analyst studies the status, conditions, and trends within a large study area. An example in a NFI includes the demographic processes of tree populations within the study area, such as total tree growth, mortality, regeneration, and removals; distribution of tree species and tree sizes; supplies of wood fiber by different merchantability categories, including forecasts of future supplies; and indicators of forest health. Other examples include landscape-level changes in the extent and composition of forest stands and plant communities according to detailed classification systems. Major disturbances and changes in land use affect these characteristics of the population, and the remote sensing technologist can detect and quantify the magnitude of certain major changes. However, there are other important driving forces, such as markets for wood fiber, air pollution, climate change, drought, disease, and the ecological processes inherent to forest communities. Compared with major changes in land cover, many demographic and landscape changes evolve slowly over time, with spatial patterns that can be diffuse and fine grained within the landscape.

The NFI resource analyst typically investigates changes in a study area at a detail well beyond the measurement capabilities of synoptic orbital sensors. Much of the information relevant to the resource analyst requires detailed measurements of individual trees and forest stands by field crews. Since it is not feasible to measure every forest stand and every tree in a large study area, those detailed field measurements are made at a sample of sites. With probability sampling, those filed measurements are sufficient to produce unbiased statistical estimates of totals and means for the entire study area. The analyst uses detailed statistical tables that quantify the status and changes within the study area; these tables can require thousands of population estimates. The analyst also uses nonlinear transformations of population statistics to estimate proportions and rates of change in the study area. The thematic maps produced by the remote sensing technologist provide a qualitative spatial context, but such sensor data serve an ancillary role for the analyst’s detailed investigations.

The sample survey statistician produces those thousands of statistical estimates for the study area with detailed field measurements from the probability sample. One important goal for the statistician is maximization of statistical efficiency [1] (p. 11, p. 65 and p. 227) and estimator accuracy and minimization of uncertainty given the constraints imposed by funding and the logistics of data processing. The statistician can use auxiliary information, primarily inexpensive sensor data, to improve population statistics. This is most often accomplished with conventional univariate sample survey methods, such as post-stratification and regression estimators.

The objective of this manuscript is to provide the remote sensing technologist a means to increase the value of sensor data to resource analysts. The related objective is to demonstrate to the sample survey statistician that multivariate model-assisted regression estimators are feasible in an official statistics program, which involves numerous study variables and numerous remotely sensed auxiliary variables. This statistical approach uses the Kalman filter (KF), which has been applied in navigation and control systems engineering for over 50 years. In the context of a sample survey, the KF is a type of multivariate regression estimator for population statistics. The KF can improve a vector of population statistics for study variables with a vector of population statistics for auxiliary variables.

The KF uses a state space representation of population parameters for the sampled population. In the context of the sample surveys, each state variable in the state vector is considered a “population parameter,” and each estimated state variable is a “population statistic.” The state vector contains all relevant population parameters. In the implementation below, one partition of the state vector contains the full time series of study variables that are important to the analyst, while another partition contains the full time series of auxiliary sensor variables. The KF constrains sample survey estimates for the auxiliary variables to exactly equal their census constants, which are known exactly from GIS enumeration of remotely sensed and other geospatial data for each and every pixel and polygon in the target population. These constraints improve statistical efficiency and reduce uncertainty for every study variable that is well correlated with any auxiliary sensor variable.

## 2. State Space

The following application of the Kalman filter (KF) uses the state space representation of a sampled population. In this context, state space is a set of statistical values, namely the “state vector,” that sufficiently quantifies all relevant attributes of that population. The state vector is denoted as **x**, and each element in the state vector is a “state variable.” Consider the case in which there are *k_s_* state variables that are important to the analyst, termed “study variables,” and *k_a_* remotely sensed state variables, termed “auxiliary variables” [1]. The latter are primarily intended to reduce uncertainty in estimates of correlated study variables.

In the following implementation of the KF, the state vector has a hierarchical structure. The first *k_s_*-by-1 partition, denoted as vector **x***_s_*, includes parameters for the study variables, while the second *k_a_*-by-1 partition, denoted as vector **x***_a_*, includes parameters for the auxiliary geospatial and remotely sensed variables. The full state vector **x** has dimensions of *k* = (*k_s_* + *k_a_*):

(1) $$x=\left[\begin{array}{l} x_{s}\\ x_{a} \end{array}\right]$$

In the case of an NFI, some state variables have area as their unit of measure, such as the total hectares for a certain category of land use in the entire study area. Other state variables have different units, such as the number of individuals in a subpopulation or the number of remotely sensed pixels in the population that are classified into a certain category. Assume conventional sample survey methods [1] make the initial estimate of the *k*-by-1 state vector in Equation (1). An example is given in Section 2.2 (below). The full state vector of population parameters is completely observable with this initial multivariate sample survey estimate.

The initial state vector has an associated *k*-by-*k* state covariance matrix for random errors that is consistent with the same design based estimator (see Section 2.2 below). The state covariance matrix quantifies the strength of linear relationships among all statistical estimates in the state vector, including those among the study variables and the auxiliary sensor variables. The *k*-by-*k* state covariance matrix has conformable partitions with the state vector in Equation (1):

(2) $$V=\left[\begin{array}{ll} V_{s} & {V^{\prime }}_{s,a}\\ V_{s,a} & V_{a} \end{array}\right]$$

where **V***_s_* is the *k_s_*-by-*k_s_* partition with the covariance matrix for the study variables; **V***_a_* is the *k_a_*-by-*k_a_* partition with covariance matrix for the auxiliary sensor variables; and **V***_s_*_,*a*_ is the *k_a_*-by-*k_s_* matrix partition of covariances between the study variables and the auxiliary sensor variables. The degree to which the KF can improve estimates of the study variables depends upon the absolute magnitude of the covariances in partition **V***_s_*_,*a*_.

### 2.1. Time Series Structure for the State Vector

In a typical engineering application, the “dynamic” KF recursively “updates” estimates of the state vector at each step *t* in the discrete time series *t* = 1, 2, …, *p*. A single “recursion” of the dynamic KF combines a model-based estimate of the state vector for time *t* with an independent observation vector for the state of that system at time *t*. This updated KF estimate for time *t* has less uncertainty (*i.e.*, smaller variance) than either the observation or the initial model based estimate for time *t*. Next, a linear model predicts the value of the state vector at time *t* + 1 given the best estimate at time *t*, and the recursive KF estimation repeats for time *t* + 1.

Unlike most applications of the KF, the following algorithm is a “static” estimator that uses only one recursion. There is no formal deterministic model that predicts the state of the system at time *t* based on the best estimate at time *t* − 1. The following algorithm implements a model-assisted estimator, not a model-based method (see [1] for definitions). However, this algorithm can accommodate dynamic time series. The leading *k_s_*-by-1 partition of the state vector can have sub-partitions for each and every discrete time *t*. For example, if there were 11 annual time steps, and estimates for a set of 50 study variables are required for each time step, then the leading partition of the state vector would have *k_s_* = 550 elements. If there is a time series of 30 auxiliary sensor variables at each of those 11 time steps, then in the trailing partition of the state vector would have *k_a_* = 330 elements, and the full state vector would have a total of *k* = 880 elements.

Let [**x***_t_*_=*j*_]*_s_* be the vector of population parameters for all study variables measured at time *t* = *j* in the time series of *p* years, beginning with year *t* = 1 and each year thereafter until year *t* = *p*:

(3) $$x=\left[\begin{array}{l} x_{s}\\ x_{a} \end{array}\right]=\left[\begin{array}{c} {\left[\begin{array}{c} x_{t=1}\\ x_{t=2}\\ \vdots \\ x_{t=p} \end{array}\right]}_{s}\\ x_{a} \end{array}\right]$$

Each temporal partition in the time series can share the same types of study variables, For example, the *i*th element of partition [**x***_t_*_=*j*_]*_s_* might be the area of the sampled population covered by forested lands at time *j*, while the *i*th element of partition [**x***_t_*_=*j+*1_]*_s_* might be the area of the sampled population covered by forested lands at time *t* = (*j* + 1). The corresponding state covariance matrix for estimation errors from the sample survey is:

(4) $$V=\left[\begin{array}{ll} V_{s} & {V^{\prime }}_{s,a}\\ V_{s,a} & V_{a} \end{array}\right]=\left[\begin{array}{ccccc} {\left[V_{t=1}\right]}_{s} & {\left[V_{t=1,t=2}\right]}_{s}^{\prime } & \cdots & {\left[V_{t=1,t=p}\right]}_{s}^{\prime } & {\left[V_{t=1,a}\right]}_{s}^{\prime }\\ {\left[V_{t=1,t=2}\right]}_{s} & {\left[V_{t=2}\right]}_{s} & \ldots & {\left[V_{t=2,t=p}\right]}_{s}^{\prime } & {\left[V_{t=2,a}\right]}_{s}^{\prime }\\ \vdots & \vdots & \ddots & \vdots & \vdots \\ {\left[V_{t=1,t=p}\right]}_{s} & {\left[V_{t=2,t=p}\right]}_{s} & \cdots & {\left[V_{t=p}\right]}_{s} & {\left[V_{t=p}\right]}_{s,a}^{\prime }\\ {\left[V_{t=1}\right]}_{s,a} & {\left[V_{t=2}\right]}_{s,a} & \cdots & {\left[V_{t=p}\right]}_{s,a} & V_{a} \end{array}\right]$$

The partition with the remotely sensed geospatial auxiliary variables **x***_a_* is treated as time invariant even though there can be time series of sensor data. This is analogous to a photographic image, which does not change even though the subject of that image can change in the future and differed in the past. An image taken at one point in time can be correlated with the state of the subject in the past, present, and future. Most likely, the correlations will be strongest between sensor measurements and field measurements where the differences in measurement times are the smallest. Covariances between the time series of study variables and the time series of auxiliary sensor variables are in the last rows and last columns partitions of the covariance matrix in Equation (4). This structure for the state vector is one of several new features in this application of the KF to time series analyses.

### 2.2. Initial Estimate for the State Vector

Initialize the application with a design-based sample survey estimate for the full state vector. This estimate serves as the initial input into the KF, *i.e.*, *t* = 1. For simplicity, assume the special case of a simple random sample in which each sampled unit is measured at each time step. This initial estimate equals the (*k_s_* + *k_a_*)-by-1 vector mean for the random sample of *n* units, where each sampled unit *i* has a (*k_s_* + *k_a_*)-by-1 vector measurement:

(5) $${\hat{x}}_{SRS}=\frac{1}{n}\sum_{i=1}^{n}{\left[\begin{array}{c} {\left[\begin{array}{c} x_{t=1}\\ x_{t=2}\\ \vdots \\ x_{t=p} \end{array}\right]}_{s}\\ x_{a} \end{array}\right]}_{i}={\left[\begin{array}{c} {\left[\begin{array}{c} {\hat{x}}_{t=1}\\ {\hat{x}}_{t=2}\\ \vdots \\ {\hat{x}}_{t=p} \end{array}\right]}_{s}\\ {\hat{x}}_{a} \end{array}\right]}_{SRS}$$

Its estimated (*k_s_* + *k_a_*)-by-(*k_s_* + *k_a_*) covariance matrix is:

(6) $$\begin{array}{c} {\hat{V}}_{SRS}=\frac{1}{n}\left[\frac{1}{n-1}\sum_{i=1}^{n}\left({\left[\begin{array}{c} {\left[\begin{array}{c} x_{t=1}\\ x_{t=2}\\ \vdots \\ x_{t=p} \end{array}\right]}_{s}\\ x_{a} \end{array}\right]}_{i}-{\left[\begin{array}{c} {\left[\begin{array}{c} {\hat{x}}_{t=1}\\ {\hat{x}}_{t=2}\\ \vdots \\ {\hat{x}}_{t=p} \end{array}\right]}_{s}\\ {\hat{x}}_{a} \end{array}\right]}_{SRS}\right){\left({\left[\begin{array}{c} {\left[\begin{array}{c} x_{t=1}\\ x_{t=2}\\ \vdots \\ x_{t=p} \end{array}\right]}_{s}\\ x_{a} \end{array}\right]}_{i}-{\left[\begin{array}{c} {\left[\begin{array}{c} {\hat{x}}_{t=1}\\ {\hat{x}}_{t=2}\\ \vdots \\ {\hat{x}}_{t=p} \end{array}\right]}_{s}\\ {\hat{x}}_{a} \end{array}\right]}_{SRS}\right)}^{\prime }\right]\\ ={\left[\begin{array}{ccccc} {\left[{\hat{V}}_{t=1}\right]}_{s} & {\left[{\hat{V}}_{t=1,t=2}\right]}_{s}^{\prime } & \cdots & {\left[{\hat{V}}_{t=1,t=p}\right]}_{s}^{\prime } & {\left[{\hat{V}}_{t=1,a}\right]}_{s}^{\prime }\\ {\left[{\hat{V}}_{t=1,t=2}\right]}_{s} & {\left[{\hat{V}}_{t=2}\right]}_{s} & \ldots & {\left[{\hat{V}}_{t=2,t=p}\right]}_{s}^{\prime } & {\left[{\hat{V}}_{t=2,a}\right]}_{s}^{\prime }\\ \vdots & \vdots & \ddots & \vdots & \vdots \\ {\left[{\hat{V}}_{t=1,t=p}\right]}_{s} & {\left[{\hat{V}}_{t=2,t=p}\right]}_{s} & \cdots & {\left[{\hat{V}}_{t=p}\right]}_{s} & {\left[{\hat{V}}_{t=p}\right]}_{s,a}^{\prime }\\ {\left[V_{t=1}\right]}_{s,a} & {\left[V_{t=2}\right]}_{s,a} & \cdots & {\left[{\hat{V}}_{t=p}\right]}_{s,a} & {\hat{V}}_{a} \end{array}\right]}_{SRS} \end{array}$$

### 2.3. Observation Vector

In addition, the KF uses auxiliary information from an independent “observation vector,” which is referred to as the “measurement vector” by some authors in the KF literature. In the following implementation, elements in this *k_a_*-by-1 vector, with its exact population means for auxiliary sensor variables as enumerated with a GIS, conform to the trailing *k_a_*-by-1 partition of the state vector in Equation (1), with its sample survey estimates for those same auxiliary sensor variables. The population census parameters for all *k_a_* auxiliary sensor variables over all *N* pixels or polygons in the statistical population are enumerated with a GIS as:

(7) $$y=\frac{1}{N}\sum_{i=1}^{N}{\left[\begin{array}{c} y_{1}\\ \vdots \\ y_{k_{a}} \end{array}\right]}_{i}$$

## 3. Kalman Filter Estimator

The following application of the KF uses complete census information about remotely sensed auxiliary variables to increase precision in estimated population statistics for the study variables. It does this by constraining the *k_a_*-by-1 partition of the state vector for the auxiliary statistics, which is estimated with the sample survey in Equation (5), to equal its exact true values in the observation vector **y***_a_* in Equation (7). This reduces variance of the estimate for any study variable that has a significant nonzero covariance with any auxiliary variable. (If the covariance with a study variable is zero, then the KF has zero effect on that study variable.) The KF applies these constraints as follows [2].

Let *k_a_*-by-*k* matrix constant **H** extract the *k_a_*-by-1 partition for the auxiliary variables from the full *k*-by-1 estimate of the state vector:

(8) $${\left[{\hat{x}}_{a}\right]}_{SRS}=H{\left[\begin{array}{c} {\hat{x}}_{s}\\ {\hat{x}}_{a} \end{array}\right]}_{SRS}$$

where **H** = [**0** | **I***_a_*], **0** is the *k_a_*-by-*k_s_* zero matrix, and **I***_a_* is the *k_a_*-by-*k_a_* identity matrix. In the KF literature, **H** is termed the “measurement matrix” or “observation matrix”.

The estimator for the state vector after the KF update [2] is the matrix-weighted sum of the *k*-by-1 vector estimate of **x***_SRS_* from the sample survey and the *k_a_*-by-1 observation vector **y***_a_* from the GIS enumeration of values for every pixel and polygon in the statistical population:

(9) $${\hat{x}}_{KF}=\left(I-W\;H\right){\hat{x}}_{SRS}+W\;y_{a}$$

where **I** is the *k*-by-*k* identity matrix. **W** is the *k*-by-*k_a_* matrix of Kalman weights, which is defined below in Equation (12). This single recursion with the KF may be considered an application of the multivariate composite estimator. If there were only one study variable, then Equation (9) is algebraically identical to the g-weighted regression estimator. The KF estimator for the state covariance matrix after the Kalman update [2] in Equation (9) is:

(10) $${\hat{V}}_{KF}=\left(I_{s+a}-W\;H\right){\hat{V}}_{SRS}{\left(I_{s+a}-W\;H\right)}^{\prime }+W\;V_{a}\;W$$

The estimated value of **V**_SRS_ in Equation (10) is the *k*-by-*k* covariance matrix from the sample survey as in Equation (6), and **V***_a_* is the *k_a_*-by-*k_a_* covariance matrix for the independent population estimates of auxiliary variables. The vector of population parameters **y***_a_* in Equation (9) is a known constant because it is the enumeration of all pixel and polygon values in the statistical population; therefore, its covariance matrix for random sampling errors is **V***_a_* = **0**, which simplifies Equation (10) to:

(11) $${\hat{V}}_{KF}=\left(I_{s+a}-W\;H\right){\hat{V}}_{SRS}{\left(I_{s+a}-W\;H\right)}^{\prime }$$

The optimal *k*-by-*k_a_* KF weight matrix **W** is computed [2] as:

(12) $$W={\hat{V}}_{SRS}H^{\prime }{\left(H{\hat{V}}_{SRS}H^{\prime }\right)}^{-1}$$

The KF weight matrix **W** in Equation (12) yields the Best Linear Unbiased Estimator (BLUE) for the state vector [2], conditional upon the estimated covariance matrix ${\hat{V}}_{SRS}$ from Equation (6). The KF estimator is “optimal” in the sense of a minimum variance or least squares estimator [2].

Unfortunately, the numerical computation in Equation (12) is notoriously unstable, especially as **V***_a_* **→** **0**. Fortunately, the engineering literature offers alternative KF algorithms with superior numerics [3,4]. I offer one such algorithm in Section 4, which assumes the special case in which the observation vector is a known census constant, *i.e.*, **V***_a_* = **0**.

## 4. Novel Kalman Filter Algorithm

In this section, I present a new algorithm for computations necessary to apply Equations (9)–(12). This algorithm solves numerical problems associated with large state vectors and rank deficient state covariance matrices. It strictly assumes the observation vector is a known constant, namely, **V***_a_* = **0** in Equation (10). Like a square root filter [3,4], this algorithm sequentially applies one auxiliary sensor variable at a time. In this way, the algorithm replaces the matrix inverse in Equation (12) with the scalar inverse. This simplifies the program code required for inversion of an ill conditioned or singular covariance matrix partition **V***_a_* in the estimated state vector; see Equation (6). However, unlike a conventional square root filter, the algorithm does not require prior orthogonalization of that matrix partition [4] (Chapter 7.4), which reduces the computational burden, especially with a large partition of the covariance matrix.

A single invocation of the algorithm iterates through *k_a_* loops in lines 3 to 15, with one iteration for each of the *k_a_* auxiliary sensor variables. Within the *i*th iteration, the KF update uses the *i*th census constant *y_i_* in Equation (7) to improve the KF estimate for the entire state vector. By “improve,” I mean reduce the magnitude of random estimation errors as measured with the covariance matrix for the estimated state vector. Each iteration improves the KF estimate for any study variable that has a nonzero covariance with the *i*th auxiliary variable after the (*i* − 1)th loop. Those covariances reside in partitions of the state vector and state covariance matrix having subscripts 1, 2, …, *k_s_*. In addition, the *i*th KF update improves estimates for the remaining auxiliary sensor variables, which reside in partitions with subscripts (*k_s_* + 1), (*k_s_* + 2), …, (*k_s_* + *i* − 1). If there is zero covariance between any one of these variables and the *i*th auxiliary variable, then the KF places zero weight on the *i*th auxiliary variable, meaning the KF has no effect on such these variables during the *i*th iteration. The KF vector estimate after the *i*th iteration serves as initial conditions for the KF in the next (*i* + 1)th iteration.

After the *i*th iteration, the KF estimate of the (*k_s_* + *i*)th state variable, which is the estimated population mean for the *i*th auxiliary sensor variable, will exactly equal its corresponding census constant *y_i_*, which is computed with the GIS enumeration in Equation (7). This means that the KF “estimate” for that state variable has no random estimation error. Therefore, the (*k_s_* + *i*)th row and column of the state covariance matrix equal the conformable zero vector in Equation (A14), which by definition makes the (*k_s_* + *i*)th state variable orthogonal to all other estimated state variables. In other words, the algorithm performs a sequential orthogonalization of the (*k_s_* + *i*)th state variable within each iteration. This is computationally more efficient, and perhaps numerically more accurate, than a square root filter that requires prior orthogonalization of the full *k_a_*-by-*k_a_* covariance matrix partition **V***_a_*. of the estimated state covariance matrix in Equation (6).

Because the algorithm uses a scalar inverse rather than a matrix inverse, it can readily accommodate an ill conditioned or singular covariance matrix. I now explain. The KF weight vector **W** from Equation (12) is computed in line 11 of the algorithm. This requires the scalar inverse of the variance on the (*k_s_* + *i*) th diagonal element of the estimated state covariance matrix from previous iteration. If that variance is very small (e.g., less than 10^−4^), then its inverse will be excessively large (e.g., greater than 10^4^). This risks numerical instability. If that variance is nearly zero, then the algorithm omits the KF constraint with the *i*th auxiliary census constant (see line 5 of Algorithm 1). An estimate of an auxiliary sensor variable with a very small variance typically has very low covariance with the study variables. Therefore, omission of such an auxiliary sensor variable will not notably sacrifice statistical efficiency.

**Algorithm 1** R code that applies sequential Kalman filter (KF) update with known observation constants (measurement vector) and rank deficient state covariance matrix.Inputx is (ks + ka)-by-1 design based initial estimate of state vectorV is (ks + ka)-by-(ks + ka) design based initial estimate of covariance matrixy is ka-by-1 conformable vector of auxiliary census constantsks is number of study variableska is number of auxiliary variablesk = ks + ka1w < − matrix(0,k,1)           # initialize with k-by-1 zero vector2I.wh < − diag(k)             # initialize with k-by-k identity matrix3for ( i in ka:1 ) {         # i = { ka, (ka − 1), ..., 1}4  k < − ks + i5  if ( V[k,k] < tol ) next # tol near zero (e.g., tol = 0.0001)6  c = 2*abs( y[i] − x[k])/V[k,k]7  if ( c > 1 ) { # divergence constraint from Equation (A17)8    V[1:k,k] < −V[1:k,k]*c9    V[k,1:k] < −V[k,1:k]*c10  } # end of divergence “if statement”11  w[1:k] < −V[1:k,k]/V[k,k]12  I.wh[1:k,k] < −(-w[1:k] ) # subtract vector w from k-th column13  x[1:k] < − ( I.wh[1:k,1:k] %*% x[1:k] ) + ( w[1:k]*y[i] )14  V[1:k,1:k] < −I.wh[1:k,1:k] %*% V[1:k,1:k] %*% t( I.wh[1:k,1:k] )15}                            # end of i-th iterationDefinitions[a:b,c] is the matrix partition, rows a to b, column c<− is the replacement operatorabs(z) is the absolute value of scalar zt(A) is the transpose of matrix A%*% is the matrix multiplication operatorw is the k-by-1 vector of computed optimal Kalman weightsI.wh is the k-by-k matrix of complementary Kalman weightsOutputx is (ks + ka)-by-1 state vector after Kalman updates with ka auxiliary variablesV is (ks + ka)-by-(ks + ka) covariance matrix with ka auxiliary variables

Successful applications of the KF require close attention to residual differences between statistical estimates of auxiliary sensor variables and their exact census values. This can be caused by extreme random sampling errors and undetected non-sampling errors, such as database or software errors. If that residual difference is unexpectedly large relative to its estimated variance, then “too much weight” can be assigned to the auxiliary sensor variable, thus causing “divergence.” An estimated variance of the residual that exceeds two standard deviation units (line 6 in the algorithm) is an indication of a significant source of non-sampling error. Whenever this occurs, I assume the *i*th row and column of the estimated covariance matrix is “too small.” To mitigate this apparent anomaly, the algorithm inflates the *i*th row and column of the estimated covariance matrix (see lines 8 and 9 in algorithm) so that the resulting residual difference is exactly two standard deviations from its estimated expectation. The mathematical statistician should refer to Appendix A.3 for details. 

The above algorithm can potentially process thousands of auxiliary sensor variables to improve estimates for thousands of study variables. But there is a risk. Consider one of many auxiliary sensor variables that truly have zero correlation with certain study variables. The true value of that correlation is unknown; only a statistical estimate of that value is known. That statistical estimate will likely be very close to zero; however, through random sampling error, that estimate will not exactly equal zero. The KF will reduce the estimated variance for every study variable that has a nonzero covariance with an auxiliary sensor variable, even if that covariance is very small. The reduction will be small, but not zero. Assume there are large numbers of auxiliary sensor variables that are truly uncorrelated with a study variable, but they have estimated covariances that are nonzero but very small. Each such auxiliary sensor variable will contribute a small reduction in the variance of the estimated study variables. This can unintentionally accumulate into a notably large reduction. Methods given in Appendix A.4 (below) mitigate this risk. Those methods identify each off-diagonal element of the state covariance matrix that has a correlation smaller than that expected by chance. Before and after invocation of the algorithm, the estimated covariance for each element that is nearly equal to zero should be changed to exactly zero.

Another risk is a large random sampling error in estimation of the correlation (covariance) between a study variable and an auxiliary sensor variable. If the known estimate substantially differs from its unknown true value, then too much or too little KF weight is placed on the auxiliary variable. Therefore, the KF should only be applied to variables with sufficient sample size. Section 5 and Appendix A use the following criterion: every study variable and auxiliary sensor variable must have a nonzero measured value for least 25 sampling units.

## 5. Case Study

**Figure 1:**
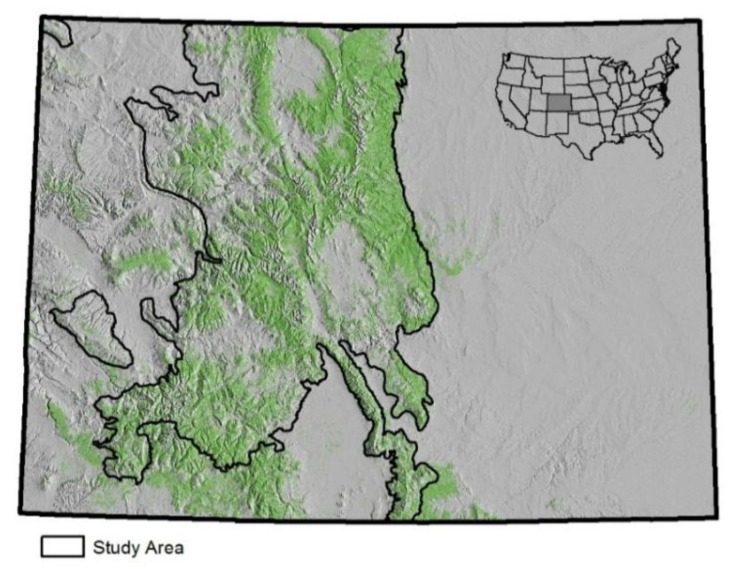
Study area (91,976 km^2^) and spatial distribution of forest cover across the State of Colorado, which covers an area 600 km east to west and 450 km north to south.

Application of the algorithm is demonstrated with the following case study. It uses NFI data from the Forest Inventory and Analysis (FIA) Program in the USA [5]. The case study includes two objectives. The first involves estimation of standard official statistics in a forest monitoring program, which is intended to evaluate the capability of the multivariate algorithm to produce large numbers of population statistics. The second objective tests application of the algorithm in the context of a special study, which is often a high priority to the resource analyst. This special study investigates an epidemic of tree mortality in the State of Colorado from mountain pine beetles (*Dendroctonus ponderosae* Hopkins), which has swept through the montane forests of the western United States during the past 10 years [6,7]. Tree mortality has been most severe in the lodgepole pine (*Pinus contorta*) tree population. This phenomenon is highly concentrated into discrete patches of extensive forests, which can be described as tree mortality at the stand scale. Colorado is among the most severely impacted geographic areas in the United States. This case study analyzes the portion of Colorado that contains the vast majority of lodgepole pine forests, regardless of current impacts from mountain pine beetles. The study area covers approximately 30% of Colorado (Figure 1). Annual FIA data are available for each of 11 years beginning in 2002.

**Figure 2:**
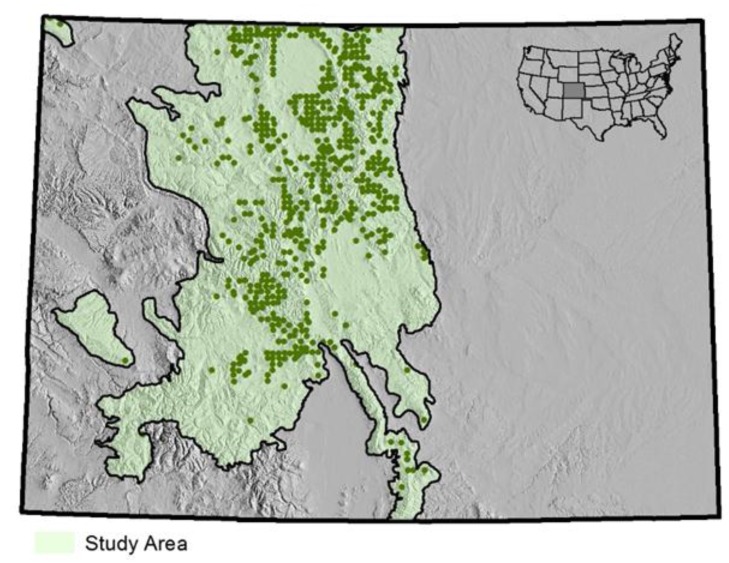
Approximate locations of FIA ground (field) plots in study area that are classified as forest with lodgepole pine trees. The figure does not identify locations for the remaining 75% of other FIA sample plots in the study area, which are distributed on an approximate 5 km by 5 km grid; however, all sampled plots in the study area, regardless of forest classification, are used in the sample survey estimators.

In order to produce standard NFI statistics, there are 3788 FIA plots in this study area, with an average of about 380 sample plots measured each year within an annual panel of primary sampling units, which is a probability sample of the entire study area. An average of 230 of these plots fall within forested conditions each year, of which an average of 60 plots have one or more lodgepole pine tally trees (Figure 2). Within any single annual panel, 43 to 60 plots had live lodgepole pine tally trees infested with insects; 17 to 30 plots had lodgepole pine mortality trees in the tally.

The case study also includes separate estimates for a small analysis area, namely the Arapaho Roosevelt National Forest (ARNF), which covers the 6% study area that is most severely impacted by the mountain pine beetle epidemic. There are 239 FIA plots in this National Forest, with an average of 24 plots measured each year. I use a three-year moving window of remeasured FIA plots to increase sample size from multiple annual FIA panels for this small area. The three-year window is a compromise between augmentation of the sample size and retention of interpretable time series estimates. The ARNF example is typical for special studies conducted by the analyst.

The statistical estimates for study variables include: basal area and numbers of lodgepole pine trees classified by their mortality status; area by forest type; stocking; net tree growth. The result is 212 study variables. However, many of those attributes are rare, even with a three-year moving window. As recommended in Section 4, any study variable having less than 25 nonzero values in the sample at any of the 11 time steps is omitted; therefore, estimates for only 136 of those 212 study variables are made with the KF.

For remotely sensed and other geospatial auxiliary variables, this case study uses a combination of: polygon data from airborne reconnaissance techniques; digital data from two orbital sensors, each with a different pixel resolution; and independent polygon data from a biogeographical model. The spatial resolution of these auxiliary data range from 0.1-ha pixels to nearly one million-hectare polygons. Each set of spatial data completely tessellates the entire study area so that the exact population parameter value for every auxiliary variable is known through enumeration of all pixels and polygons with a GIS (Section 2.3).

One set of auxiliary variables uses geographic data from Aerial Detection Surveys (ADS), which are conducted each year by the Forest Health Protection Program. While systematically flying in light aircraft over impacted forests, trained ADS observers visually map broad areas based on visual evidence of episodic tree mortality, where each mapped polygon can represent hundreds of hectares. An ADS “mortality polygon” circumscribes an area that contains obvious concentrations of tree mortality. Each ADS mortality polygon ranges in size from 5 to 500 ha. However, each mortality polygon is a heterogeneous mixture of impacted forest, unaffected forest, and non-forest. Other polygons record areas that were surveyed by ADS observers but contained no remarkable areas of tree mortality. All remaining portions of the study area are classified as unobserved, which typically connotes low impacts from insect damage. There is variability in classifications among observers. These three categories of ADS classifications are available in the GIS for each of the 19 years from 1999 to 2012. After the transformation of these 19 multinomial variables, each with three categories, there are 57 ADS auxiliary variables.

A second set of auxiliary variables comes from multispectral data captured with the MODIS sensor aboard the Terra (EOS AM-1) and Aqua (EOS PM-1) spacecraft, which orbit 705 km above the earth’s surface. MODIS measures 36 different spectral bands, two of which have a nominal spatial resolution of 250-m (6.0-ha) pixels. There are approximately 1.5 million MODIS pixels in my study area. Remote sensing technologists developed statistical models that predict for each MODIS pixel whether or not forest cover is the predominant land cover [8].

A third set of auxiliary variables originated with the Enhanced Thematic Mapper Plus (ETM+) sensor aboard Landsat satellites, which also orbit 705 km above the earth surface. The EMT+ sensor measures seven different spectral bands, six of which have a nominal spatial resolution of 30 × 30 m (0.1-ha) pixels. There are approximately 90 million EMT+ pixels in my study area. A multiagency consortium of LANDFIRE technologists developed nonparametric statistical models that predict for each ETM+ pixel the predominant category of vegetation [8,9]. There are seven categories of vegetation, one of which is “coniferous forest,” for a total of seven binary auxiliary variables.

The ADS protocol is designed to identify “hot spots” of tree mortality each year at the landscape scale; it is not specifically designed to classify episodic tree mortality at the spatial scale of a FIA plot. Therefore, an ADS polygon is relatively large and heterogeneous, with a mixture of many forest stands among which severity of tree mortality varies, and each ADS polygon has non-forest inclusions. I attempt to improve spatial resolution of the ADS data through an intersection of the relatively large ADS polygons with the 30 × 30 m LANDFIRE pixels classified as coniferous forest. This improves agreement between ADS classifications and field observations of tree mortality. However, there are misclassifications of coniferous forest with LANDFIRE pixels, and many forested portions of an ADS mortality polygon are uninfected. Therefore, the agreement is somewhat weak among these auxiliary variables and those study variables related to individual tree mortality. Regardless, this intersection increases the number of binary ADS auxiliary variables from 57 to 114.

A fourth set of auxiliary variables comes from classifications of broad landscapes into different ecoregions based on differences in climate, land form, land management, and ecosystems [10]. I use the Subsection level in this hierarchical classification system. Subsections completely tessellate the study area into 31 binary auxiliary variables, one for each unique Subsection. The average size of a Subsection polygon is almost 1 million hectares.

Another set of auxiliary variables divides the study area based on administrative units for land management, primarily the six different National Forests in Colorado. This forces the total estimated area for each National Forest to exactly match the official administrative statistics. It also captures certain differences in forests caused by regional epidemiological conditions and land management practices. The Arapaho Roosevelt National Forest is one of those administrative units. This National Forest has experienced the most severe impacts from bark beetle epidemic in Colorado, and additional statistical estimates are made specifically for this small domain.

The final set of auxiliary variables are counties, which are administrative units for local governmental organizations. A county is a spatially contiguous geopolitical subdivision of a state. There are 3007 counties in the USA, and the average size of each is about 3000 km^2^. The area of each is an administrative census constant. Inclusion of counties as auxiliary variables is intended to assure that the total estimated area of each county agrees with its official census area, which is a common expectation from users of these statistics. The intent is not variance reduction. However, county boundaries also tessellate the study area into contiguous areas, which might capture some of the underlying geospatial patterns in the study variables. Each county is separated into two categories with 6.0-ha MODIS pixels, each of which is classified into forest cover or non-forest cover with sensor data. Since there are 46 counties, this creates 92 binary auxiliary variables in the state vector.

The outcome is a total of 252 remotely sensed and other geospatial auxiliary variables for millions of pixels and polygons. The GIS extracts the values for each of those 252 auxiliary variables for each of those 3788 plots in this case study. The GIS enumerates all pixels and polygons for those 252 auxiliary census values across the entire study area. Therefore, the statistical estimator requires a very small fraction of the spatial data processed by the GIS.

An auxiliary variable can be rare, which in my case means most plots have a measured value of zero for that variable. However, through chance alone, a rare auxiliary variable can be highly correlated with a study variable. Such an auxiliary variable would greatly reduce the estimated variance of the study variable even though there is truly little association with the auxiliary. To mitigate this risk, only those variables with non-zero measurements at 25 or more sampling units are estimated with the KF (see Appendix A.4 for details). This reduced the number of auxiliary variables from 252 to 164. Furthermore, the algorithm omits auxiliary variables whenever their estimated variance is or becomes nearly zero during each *i*th loop iteration within the algorithm. This effectively eliminates redundant information among the auxiliary sensor variables.

The case study compares improvements in the quality of monitoring data with the KF to improvements with the conventional post-stratification estimator. A typical analytical study might use post-stratification with any of the available remotely sensed data. However, the feasible number of strata is limited by the relatively small sample size of FIA plots in each annual sample panel. The stratification is based on intersection of the three different categories used in each of the annual Aerial Detection Surveys (ADS) with the time invariant LANDFIRE ETM+ classification of conifer forest. Therefore, there are six potential strata for each of the 10 FIA panels. Strata change from year to year because the remotely sensed ADS data change each year. I extrapolate the criterion offered by [11]. If a stratum during one year has fewer than 10 plots, then that stratum is combined with a similar stratum. In most cases, this meant use of a single stratum for ADS mortality polygons rather than an intersection of ADS polygons with the LANDFIRE classification of coniferous forest cover. This process reduced gains from post-stratification, but there is little choice given the limited number of available sample plots.

Post-stratification is compared with two different sets of estimates with the KF. The first set uses only ADS classifications of tree mortality intersected with LANDFIRE predictions of coniferous forest cover for ETM+ pixels. This allows direct comparison of the two post-stratification and Kalman estimators given approximately the same stratification variables and auxiliary variables. The comparison is not exact because the KF uses all 44 ADS auxiliary variables across all years, while the number of plots in each panel restricts post-stratification to ADS data from a single year. The second set of estimates with the KF uses those same 44 ADS variables plus the additional 120 auxiliary variables described above.

Table 1 summarizes the results for this case study. I use the “design effect” to compare statistical efficiency of each set of estimates to the simple random sampling estimator. The design effect [1] (p. 53) compares the efficiency of each set of estimates with the post-stratification and KF estimators to the simple random sampling estimator. A “design effect” of 10% means a 10% reduction in estimated variance relative to the variance from the simple random sampling estimator. This is an increase in statistical efficiency. A negative design effect for the post-stratification estimator means that it actually degrades estimation accuracy and reduces statistical efficiency. There are 11 estimates for each study variable, one for each year from 2002 to 2012. I present only the minimum and maximum statistics among each of those 11 years to reduce cluttered details.

The post-stratification estimator reduced statistical efficiency in this particular case study; the simple random sampling estimator has less variance in estimation errors than the post-stratification estimator for certain study variables. There are at least three potential causes for this situation in this single case study. First, post-stratification on ADS polygons can increase the “within stratum” variance for some study variables between values at sampling units and their strata means. This might be caused by the coarse spatial resolution of ADS polygons relative to the size of FIA plots, especially for panels with a sample size too small to subdivide large heterogeneous ADS polygons by LANDFIRE classifications of coniferous forest. The most useful auxiliary information from ADS polygons was identification of forests that have no evidence of insect infestations. Second, the estimated stratum mean can have substantial random sampling error, which can inflate the variance of the residual differences between values at sampling units and their estimated strata means [1] (p. 267). Third, the variance computation for post-stratification contains an adjustment for the random sample size within each stratum, and that approximation might not be sufficiently accurate given the small sample sizes in many strata.

In contrast, the KF always improves accuracy. This mathematical axiom is apparent from Equations (A8) and (A15) in the Appendix A (below). The improvements are generally greatest in the estimates for the Arapaho Roosevelt National Forest with the study variables for infestation and tree mortality. This National Forest has few FIA plots compared to the entire study area. The KF was especially effective for small domains in this particular case study.

If attention were restricted to those estimation years with the maximum improvement from post-stratification, then improvements from post-stratification are somewhat greater than most improvements with the KF, at least when they share the same post-stratification and auxiliary variables. However, the pattern is reversed with the KF that uses all auxiliary variables, regardless of whether or not they are used for post-stratification. The KF generally produces more precise and reliable estimates than post-stratification in this one case study. However, this does not necessarily mean the KF will be more efficient than post-stratification in other studies.

**Table 1:** Change in statistical efficiency with auxiliary sensor data. The minimum and maximum statistics among 11 years are presented. Negative values indicate a loss of statistical efficiency.

Small-Area (Arapaho Roosevelt NF) Estimates for Three-Year Moving Window	Minimum Efficiency Gain or Loss			Maximum Efficiency Gain or Loss		
	Post-Stratification Estimator	Kalman Filter, Post-Stratification Auxiliary Variables Only	Kalman filter, All Auxiliary Variables	Post-Stratification Estimator	Kalman Filter, Post-Stratification Auxiliary Variables Only	Kalman Filter, All Auxiliary Variables
Number of live uninfested lodgepole pine trees	−2.4%	1.6%	3.2%	4.4%	3.7%	6.6%
Number of live infested lodgepole pine trees	−0.7%	2.0%	4.5%	8.7%	4.9%	9.1%
Number of first-year morality lodgepole pine trees	−8.0%	0.5%	2.5%	0.2%	2.0%	7.8%
Area of forest stands dominated by large trees	−42.1%	1.6%	6.2%	−29.0%	4.1%	11.3%
Entire population, estimates for 3-year moving window						
Number of large live uninfested lodgepole pine trees	−58.3%	0.2%	1.6%	1.3%	1.4%	3.4%
Number of large live infested lodgepole pine trees	1.0%	1.5%	3.6%	8.3%	4.5%	7.4%
Number of large first-year morality lodgepole pine trees	−12.1%	0.7%	2.6%	4.0%	3.2%	6.0%
Number of small live uninfested lodgepole pine trees	−0.2%	1.1%	2.4%	5.9%	3.4%	6.1%
Number of small live infested lodgepole pine trees	−2.0%	1.2%	2.4%	9.3%	3.6%	5.4%
Number of large first-year morality lodgepole trees	−10.5%	1.0%	2.7%	2.6%	2.9%	8.3%
Area of non-stocked forest stands	−23.9%	0.4%	2.6%	−13.8%	0.7%	3.5%
Entire population, annual estimates						
Total basal area of lodgepole pine trees	1.8%	1.0%	2.2%	11.5%	3.0%	4.6%
Total net tree growth	−1.2%	0.5%	2.2%	5.0%	2.5%	4.0%
Percent tree stocking of forest stands	1.2%	0.9%	3.5%	8.6%	1.8%	5.3%

## 6. Discussion

Increases in statistical efficiency with auxiliary remotely sensed data will vary among different studies. The discussion begins with candidate sources of this variation and methods that improve efficiency. It then covers advantages of multivariate estimators such as the KF. It follows with discussion of methods that improve statistical reliability. It ends with thoughts for future research and development.

First, I extrapolate previous recommendations for stratification by [12]. The agreement between a categorical auxiliary variable (e.g., forest cover) and its categorical study variable (e.g., forest land use) should generally exceed 90% for common categories and 75% for rare categories [12]. In detailed classification systems, which have many categories, most categories are relatively rare.

Gains from multispectral remotely sensed auxiliary data depend on the strength of the linear relationships between the auxiliary variables and study variables. However, that relationship can be nonlinear. Exploratory statistical analyses can detect such relationships. A nonlinear or nonparametric statistical model can be built with remotely sensed variables to predict field measurements of study variables. Examples include k-Nearest Neighbor and [13,14,15] and Classification and Regression Trees [16]. The linear relationship between a study variable and its expected value as predicted with a nonlinear model can be more effective than direct use of remotely sensed variables as predictors. However, predictive models fit with sample survey data for training should be used with caution in estimation applications because they are not independent ancillary data, and this poses challenges akin to endogenous post-stratification [17,18].

Categorical auxiliary variables have the values of 0 or 1 for each pixel, polygon, or population unit. This produces relatively large variances around mean prevalence. Unless the distribution is extremely skewed, continuous remotely sensed variables tend to have smaller variances around their expected values. For example, a statistical model that uses remotely sensed data to predict the proportion of crown cover for each predominant tree species in each pixel might increase efficiency more than a model that classifies that pixel into a single forest type. For continuous variables, consider the recommendation by [1] (p. 224 and p. 250): the absolute value of the linear correlation between an auxiliary variable and a study variable should be 0.5 or higher. An eight-fold gain in statistical efficiency is possible with a correlation of 0.95 [1].

Because the numerics of the algorithm can accommodate very large sets of auxiliary variables, classification algorithms with remotely sensed data can be specialized for each individual category for a multinomial study variable, such as different types of land cover. For example, a separate binomial thematic map may be optimized for each individual category of land cover (e.g., one for deciduous forest cover, another for coniferous forest, and yet another for cropland). With separate thematic coverage for each individual category, a pixel need not be classified into one and only one category among multiple classification systems. However, inclusion of more auxiliary variables does not necessarily contribute more auxiliary information.

A relatively small number of outlier predictions, or a relatively small proportion of misclassified pixels, can markedly reduce the linear correlations between auxiliary sensor variables and the analyst’s study variables. This would reduce the value of auxiliary sensor data for improvements in statistical monitoring. Therefore, a remotely sensed category should not mix pixels that can be reliably classified with other pixels that are difficult to accurately classify. Following recommendations by [12], a pixel should not be assigned to a category unless there is relatively high confidence that the remotely sensed classification agrees with the classification field protocol. For example, time series of remotely sensed classifications can identify geographic areas affected by rapid changes in forest cover over time. Remotely sensed data gathered before those changes might be poorly correlated with field observations after those changes. Another example is pixels affected by challenging conditions, such as cloud shadows, steep slopes, sparsely stocked forest, and forest stands with diverse conditions; these pixels do not necessarily need to be classified into any remotely sensed category. Misregistration between the geographic location of a plot and its corresponding pixel or polygon degrades associations among auxiliary sensor variables and field measurements of study variables, especially in heterogeneous, fine-grained landscapes. Methods that might reduce this source of non-sampling error are reviewed by [2] (p. 100).

The case study includes multivariate vector estimates that improve standard official statistics, such as detailed statistical tables [19]. The state vector can include a study variable for each and every cell in every statistical table. The row and column margins of each table can be the postestimation sum of estimates for each cell, and the estimated variances for each statistic on the margin computed with the full state covariance matrix [2]. This would assure that sums of estimated cell values in statistical tables agree with their corresponding row and column totals, including consistency among variance estimates.

The vast majority of sample survey estimators are designed or optimized for a single univariate study variable. Large numbers of estimates produced by official statistics organizations are typically a collection of multiple univariate estimates. Each has an estimated variance for estimation errors, but the sample survey literature rarely considers the covariance matrix for joint estimation errors among numerous population statistics. Off-diagonal covariances serve an important role in variance estimation for transformations of population estimates that are important to analysts. An example is a simple linear transformation of estimates, such as the marginal sum of cells in an estimated statistical table [1] (p. 399). An example of a nonlinear transformation is the proportion of all live lodgepole pine trees that are heavily infested with bark beetles at time *t*. This is the ratio of the estimated number of infested trees divided by the estimated total number of live trees. Yet another example is the mortality rate, which is defined as the number of live infested trees at time *t* divided by the number of recent mortality trees at time *t* + 1. In a NFI panel design, the same sample tree is never measured at both times *t* and (*t* + 1) because each NFI plot is measured only once every 5 to 10 years. Therefore, estimates for annual mortality rates are feasible only at the population level. These transformations require linear or nonlinear transformations of elements in the estimated state vector, and variance estimates for these transformations all require estimates for the covariances among study variables.

Transformations of the state vector are known as “synthetic estimators” and “pseudo estimators” in the sample survey literature [1] (pp. 173−174, pp. 205−207 and p. 399). The associated variance estimators require complementary transformations of the state covariance matrix. For example, [2] (pp. 68−87) creates a new postestimation state variable for each synthetic and pseudo estimator and develops multivariate linear transformations that appends those to the state vector and state covariance matrix. That covariance matrix has sufficient partitions for all study variables after the Kalman update plus additional partitions for all postestimation synthetic and pseudo estimates. This facilitates additional transformations of synthetic and pseudo estimates; an example is the estimated difference in annual survival rates between large and small trees. Therefore, the analyst may transform multiple time series of statistical estimates for the entire population, small areas or small domains through multiple sequential combinations of the addition, subtraction, multiplication and division operators. Variance estimators for all of these transformations are available in [2].

A large number of auxiliary variables is analogous to a large number of predictor variables in a multiple regression. There are risks from outliers, chance correlations, numerical errors, estimates for rare variables that are nearly sampling zeroes, and ill-conditioned partitions for auxiliary variables in the state covariance matrix. These risks are mitigated in multiple ways.

1. Analyze only those study variables and auxiliary variables that have nonzero values for 25 or more sampling units.
2. The algorithm in Section 4 (see line 5) uses a scalar inverse, which avoids numerical problems with the matrix inverse in Equation (12).
3. The algorithm (see lines 6 to 10) uses the constraint from Appendix A in Equation (A17), which hedges against a suspiciously large residual difference between the populations estimate for an auxiliary variable and its true census value. Such outliers can be a result of non-sampling errors, or rare random sampling errors, or random error in estimates of the covariance between study variables and auxiliary variables, or an auxiliary variable that is highly correlated with other auxiliary variables that were previously processed by the algorithm.
4. Use statistical tests to identify very small correlations between auxiliary sensor variables and study variables that are likely caused by random chance (Appendix A.4). Re-estimate those correlations as zero rather than the small nonzero value. This mitigates unintended cumulative consequences with large numbers of auxiliary variables, each of which is well correlated with certain study variables but has nearly zero correlation with other study variables.
5. To further reduce potential numerical errors, center state space on the zero vector, and subsequently scale state space so that the covariance matrix from the multivariate design based estimator has the unit diagonal. After the KF estimation process is complete, re-transform state space back to its original metrics.

Finally, the analyst must be alert for apparent anomalies among estimates. These can be obvious during careful comparisons of different estimates, as in a time series graph for a study variable. In aggregate, I believe these measures can reasonably assure the algorithm is robust to anomalies in a realized sample, non-sampling errors, and numerical errors.

I urge caution in extrapolating results from the case study in Section 5. That case study uses a single realization of a single sample from a single geographic area with a single set of auxiliary variables. Different populations with different auxiliary sensor variables might be more, or less, correlated with different study variables. Additional case studies would provide stronger conclusions regarding the post-stratification and Kalman estimators.

## 7. Conclusions

Demand for reliable NFI statistics to monitor forests and other environments is increasing, while the available resources become more constrained. Conventional sample survey methods, such as those deployed by an NFI, are successful for monitoring at the national and regional extents, but they become less accurate as the size of a study area, and the corresponding sample size, become smaller. Remotely sensed and other geospatial data can improve monitoring information and statistical efficiency at relatively little incremental cost, especially with time series estimates for small areas and small domains. The degree of improvement depends on the degree of association and correlation among auxiliary sensor variables and the analysts’ study variables.

In concert, these methods and the algorithm offered here provide an efficient and relatively simple approach to multivariate time series analyses with complex sample surveys, panel designs, sampling unit configurations, measurement systems, and auxiliary sensor data. They support pseudo estimators for linear and nonlinear transformations of population statistics, including their variance and covariance estimators [1,2]. An example is the differences in annual tree survival rates among different small areas. These types of monitoring statistics, which extend well beyond tables of summary statistics, are important to analysts.

I develop a simple, robust and efficient algorithm for the Kalman update. This algorithm has the potential to improve costly sample surveys with relatively inexpensive remotely sensed auxiliary data. It is based on a state space perspective that is familiar in the engineering literature but is rarely applied in the statistical sample survey literature. To the best of my knowledge, the algorithm reported here is a new contribution. I expect this contribution will increase the value of sensor data to programs that produce official statistics, such as an NFI. However, most important, better integration of remote sensing with field surveys holds considerable potential to improve the monitoring and management of natural resources.

## Appendix A

This section explains in detail the foundation of the Kalman filter (KF) algorithm in Section 4 (above). Appendix A.1 illustrates a simple case, in which there is one study variable and one auxiliary sensor variable, such as wall to wall full coverage pixel data from an orbital earth observing satellite sensor. Appendix A.2 extends this estimator to the case in which there is a single auxiliary sensor variable but multiple study variables. The algorithm in Section 4 sequentially applies the estimator in Appendix A.2, one auxiliary variable at a time. Appendix A.3 develops constraints to mitigate the influence of “divergent outliers”; this is implemented with lines 7 to 10 of the algorithm in Section 4. Appendix A.4 discusses a method that eliminates influence of suspected chance correlations between study variables and large numbers of auxiliary sensor variables.

### A.1. One Study Variable and One Auxiliary Variable

Consider a simple example in which there is one study variable (*k_s_* = 1), such as the area of “forest land use” as classified by a field crew, and one auxiliary variable (*k_a_* = 1), such as the remotely sensed area of “forest cover” as predicted by a model with sensor pixel data. The classifications with these two measurement protocols typically agree for about 85- to 95-percent of sample plots.

The example begins with the sample survey estimator for a simple random sample [1], which is given in Equation (A1); it produces the initial 2-by-1 vector estimate (*k* = 2) for input into the KF. There are *n* plot records, one for each sample plot. The vector measurement for the *i*th plot has two elements: (1) the proportion of the *i*th sample plot classified as forest land use with the protocol applied by the field crew; and (2) the binary 0−1 datum for classification of the *i*th sample plot regarding forest land cover with the sensor data:

(A1) $${\hat{x}}_{SRS}=\frac{1}{n}\sum_{i=1}^{n}{\left[\begin{array}{l} x_{k-1}\\ x_{k} \end{array}\right]}_{i}={\left[\begin{array}{c} {\hat{x}}_{k-1}\\ {\hat{x}}_{k} \end{array}\right]}_{SRS}\text{ where }\{\begin{array}{c} \begin{array}{l} k=2\\ 0\leq {\left[x_{k-1}\right]}_{i}\leq 1 \end{array}\\ {\left[x_{k}\right]}_{i}\in \left\{0,1\right\}\\ 0\leq {\hat{x}}_{k-1}\leq 1\\ 0\leq {\hat{x}}_{k}\leq 1 \end{array}$$

The estimator for 2-by-2 covariance matrix for random estimation errors in the sample survey [1] is:

(A2) $$\begin{array}{c} {\hat{V}}_{SRS}=\frac{1}{n}\left[\frac{1}{n-1}\sum_{i=1}^{n}\left({\left[\begin{array}{l} x_{k-1}\\ x_{k} \end{array}\right]}_{i}-{\left[\begin{array}{c} {\hat{x}}_{k-1}\\ {\hat{x}}_{k} \end{array}\right]}_{SRS}\right){\left({\left[\begin{array}{l} x_{k-1}\\ x_{k} \end{array}\right]}_{i}-{\left[\begin{array}{c} {\hat{x}}_{k-1}\\ {\hat{x}}_{k} \end{array}\right]}_{SRS}\right)}^{\prime }\right]\\ =\left[\begin{array}{ll} {\left[{\hat{V}}_{\left(k-1\right),\left(k-1\right)}\right]}_{SRS} & {\left[{{\hat{V}}^{\prime }}_{k,\left(k-1\right)}\right]}_{SRS}\\ {\left[{\hat{V}}_{k,\left(k-1\right)}\right]}_{SRS} & {\left[{\hat{V}}_{k,k}\right]}_{SRS} \end{array}\right]\text{ where }k=2 \end{array}$$

The sample survey estimates in Equations (A1) and (A2) serve as the initial state vector and its covariance matrix for the KF. In this example, the first element of the state vector contains the sample survey estimate for the area of forest land use in the population as defined by the field protocol. The second element of this vector contains the estimated area of forest cover in the population as measured with the remote sensing protocol. These initial estimates for both elements of the state vector include random sampling errors.

In addition, the population value for the second element of the state vector, which is the area of forest cover in the population as measured with the remote sensing protocol, is known exactly through GIS enumeration of all pixels in the statistical population. It equals the sum of the binary datum for the sensor classification of forest cover at each of *N* equal-sized pixels in the sampled population divided by the total number (*N*) of pixels in that population:

(A3) yk=(∑i=1N[xk]i/N)

The expected value over all possible samples for the second element in the estimated state vector in Equation (A1) equals the GIS enumeration in Equation (A3):

(A4) $$E{\left[{\hat{x}}_{k}\right]}_{SRS}=y_{k}$$

The exact population parameter *x_k_* = *y_k_* is known, but only a sample survey estimate of forest land use *x*_(*k*−1)_ is available.

The first element in the 1-by-2 vector **h** in Equation (8) equals scalar 0, and the second element equals 1. The vector multiplication of the 2-by-1 state vector **x** times the 1-by-2 vector **h** equals the sample survey estimate for the proportion of the population classified as forest land cover with remotely sensed data from the orbital satellite:

(A5) $${\left[{\hat{x}}_{k}\right]}_{SRS}=\left(h\;{\hat{x}}_{SRS}\right)=\left[\begin{array}{cc} 0 & 1 \end{array}\right]\left[\begin{array}{c} {\left[{\hat{x}}_{k-1}\right]}_{SRS}\\ {\left[{\hat{x}}_{k}\right]}_{SRS} \end{array}\right]\text{ where }k=2$$

The 2-by-1 weight vector **w** from the Kalan update is computed from Equation (12) as:

(A6) w=(V^SRSh′)(h V^SRSh′)−1=[([V^k,(k−1)]SRS[V^k,k]SRS)1]

The Kalman update in Equation (9) for the 2-by-1 state vector is:

(A7) $$\begin{array}{c} {\hat{x}}_{KF}=\left(I_{k}-wh\right)\left[\begin{array}{c} {\left[{\hat{x}}_{s}\right]}_{SRS}\\ {\left[{\hat{x}}_{a}\right]}_{SRS} \end{array}\right]+w\;y_{a}\\ =\left[\begin{array}{cc} 1 & -w_{k-1}\\ 0 & 0 \end{array}\right]\left[\begin{array}{c} {\left[{\hat{x}}_{s}\right]}_{SRS}\\ {\left[{\hat{x}}_{a}\right]}_{SRS} \end{array}\right]+\left[\begin{array}{c} w_{k-1}\\ 1 \end{array}\right]y_{a}\\ \text{=}\left[\begin{array}{c} {\left[{\hat{x}}_{s}\right]}_{SRS}+\left(\frac{{\left[{\hat{V}}_{k,\left(k-1\right)}\right]}_{SRS}}{{\left[{\hat{V}}_{k,k}\right]}_{SRS}}\right)\left(y_{a}-{\left[{\hat{x}}_{k}\right]}_{SRS}\;\right)\\ y_{a}\; \end{array}\right] \end{array}$$

Therefore, the Kalman estimate for the proportion of the sampled population classified as “forest land use” with the field protocol equals its estimate from the simple random sample plus the weighted residual difference between the exact value of the remotely sensed proportion of forest cover from the enumeration and its estimated value from the simple random sample. The “estimate” for the auxiliary variable after the Kalman update exactly equals its known population parameter value from the enumeration in the GIS (*y_a_*).

The estimated state covariance matrix after the Kalman update is computed from Equation (12):

(A8) $$\begin{array}{c} {\hat{V}}_{KF}=\left(I-wh\right){\hat{V}}_{SRS}{\left(I-wh\right)}^{\prime }\\ =\left[\begin{array}{cc} 1 & -w_{k-1}\\ 0 & 0 \end{array}\right]\left[\begin{array}{cc} {\left[{\hat{V}}_{k-1,k-1}\right]}_{SRS} & {\left[{\hat{V}}_{k-1,k}\right]}_{SRS}\\ {\left[{\hat{V}}_{k-1,k}\right]}_{SRS} & {\left[{\hat{V}}_{k,k}\right]}_{SRS} \end{array}\right]\left[\begin{array}{cc} 1 & 0\\ -w_{k-1} & 0 \end{array}\right]\\ =\left[\begin{array}{cc} {\left[{\hat{V}}_{k-1,k-1}\right]}_{SRS}-{\left[{\hat{V}}_{k-1,k}\right]}_{SRS}w_{k-1} & 0\\ 0 & 0 \end{array}\right]\\ =\left[\begin{array}{cc} \left({\left[{\hat{V}}_{k-1,k-1}\right]}_{SRS}-\frac{{\left[{\hat{V}}_{k,\left(k-1\right)}\right]}_{SRS}^{2}}{{\left[{\hat{V}}_{k,k}\right]}_{SRS}}\right) & 0\\ 0 & 0 \end{array}\right]\text{ where }k=2 \end{array}$$

The magnitude of the reduction in variance with the Kalman update depends upon the relative strength of the covariance ${\left[{\hat{V}}_{k,\left(k-1\right)}\right]}_{SRS}$. For example, the Kalman update is no more efficient than the simple random sampling estimator if there is no association between the field measurements of “forest land use” and the remotely sensed classification of “forest cover”, that is if ${\hat{V}}_{k,\left(k-1\right)}=0$ in Equation (A8). If the association were perfect, then the proportion of “forest land use” in the population would be known perfectly (*i.e*., without sampling error) because ${\left[x_{k-1}\right]}_{i}={\left[x_{k}\right]}_{i}$ for all *i* in Equation (A8). In most cases,$0<\left|\;{\left[V_{k,\left(k-1\right)}\right]}_{SRS}\right|<{\left[{\hat{V}}_{k,k}\right]}_{SRS}$ and ${\left[{\hat{V}}_{\left(k-1\right),\left(k-1\right)}\right]}_{KF}<{\left[{\hat{V}}_{\left(k-1\right),\left(k-1\right)}\right]}_{SRS}$ in Equation (A8).

### A.2. Extension to Multiple Study Variables with One Auxiliary Variable

In this section the previous results are expanded to the more general case, for which the number of study variables is *k_s_* ≥ 2. These multiple study variables remain in the leading partition, namely elements 1 through *k* − 1, while the sole auxiliary variable is in the last element *k*.

Assuming a simple random sample, the multivariate sample survey estimate for the *k_s_* study variables plus the one auxiliary variable is:

(A9) $${\hat{x}}_{SRS}=\frac{1}{n}\sum_{i=1}^{n}{\left[\begin{array}{c} \left[\begin{array}{c} x_{1}\\ \vdots \\ x_{k_{s}} \end{array}\right]\\ x_{k} \end{array}\right]}_{i}={\left[\begin{array}{c} \left[\begin{array}{c} {\hat{x}}_{1}\\ \vdots \\ {\hat{x}}_{k_{s}} \end{array}\right]\\ {\hat{x}}_{k} \end{array}\right]}_{SRS}\text{ where }k=k_{s}+1$$

with its estimated covariance matrix:

(A10) $$\begin{array}{c} {\hat{V}}_{SRS}=\frac{1}{n}\left[\frac{1}{n-1}\sum_{i=1}^{n}\left({\left[\begin{array}{c} \left[\begin{array}{c} x_{1}\\ \vdots \\ x_{k_{s}} \end{array}\right]\\ x_{k} \end{array}\right]}_{i}-{\left[\begin{array}{c} \left[\begin{array}{c} {\hat{x}}_{1}\\ \vdots \\ {\hat{x}}_{k_{s}} \end{array}\right]\\ {\hat{x}}_{k} \end{array}\right]}_{SRS}\right){\left({\left[\begin{array}{c} \left[\begin{array}{c} x_{1}\\ \vdots \\ x_{k_{s}} \end{array}\right]\\ x_{k} \end{array}\right]}_{i}-{\left[\begin{array}{c} \left[\begin{array}{c} {\hat{x}}_{1}\\ \vdots \\ {\hat{x}}_{k_{s}} \end{array}\right]\\ {\hat{x}}_{k} \end{array}\right]}_{SRS}\right)}^{\prime }\right]\\ ={\left[\begin{array}{cccc} {\hat{V}}_{1,1} & \cdots & {\hat{V}}_{1,k_{s}} & {\hat{V}}_{1,k}\\ \vdots & \ddots & \vdots & \vdots \\ {\hat{V}}_{k_{s},1} & \cdots & {\hat{V}}_{k_{s},k_{s}} & {\hat{V}}_{k_{s},k}\\ {\hat{V}}_{k,1} & \cdots & {\hat{V}}_{k_{s},k} & {\hat{V}}_{k,k} \end{array}\right]}_{SRS} \end{array}$$

In this special case, the measurement matrix **H** in Equation (8) is the 1-by-*k* row vector **h**, in which *h_i_* = 0 for 1 < *i* ≤ (*k*−1) and *h_k_* = 1.

The optimal weight matrix in Equation (12) is the *k*-by-1 column vector **w**:

(A11) $$\begin{array}{c} w=\left({\hat{V}}_{SRS}h^{\prime }\right){\left(h{\hat{V}}_{SRS}h^{\prime }\right)}^{-1}\\ =\left({\left[\begin{array}{cccc} {\hat{V}}_{1,1} & \cdots & {\hat{V}}_{1,k_{s}} & {\hat{V}}_{1,k}\\ \vdots & \ddots & \vdots & \vdots \\ {\hat{V}}_{k_{s},1} & \cdots & {\hat{V}}_{k_{s},k_{s}} & {\hat{V}}_{k_{s},k}\\ {\hat{V}}_{k,k} & \cdots & {\hat{V}}_{k_{s},k} & {\hat{V}}_{k,k} \end{array}\right]}_{SRS}\left[\begin{array}{c} 0\\ \vdots \\ 0\\ 1 \end{array}\right]\right){\left[{\hat{V}}_{k,k}\right]}_{SRS}^{-1}\\ ={\left[\begin{array}{c} {\hat{V}}_{1,k}\\ \vdots \\ {\hat{V}}_{k-1,k}\\ {\hat{V}}_{k,k} \end{array}\right]}_{SRS}\left(\frac{1}{{\left[{\hat{V}}_{k,k}\right]}_{SRS}}\right) \end{array}$$

The matrix inversion for the general case in Equation (12) is simply the scalar inversion for the special case of *k_a_* = 1 in Equation (A11). The complementary *k*-by-*k* weight matrix (**I − wh**) from Equations (9) and (11) has the following structure:

(A12) $$\left(I-wh\right)=\left[\begin{array}{ccccc} 1 & 0 & \cdots & 0 & -w_{1}\\ 0 & 1 & \ldots & 0 & -w_{2}\\ \vdots & \vdots & \ddots & \vdots & \vdots \\ 0 & 0 & 0 & 1 & -w_{k-1}\\ 0 & 0 & 0 & 0 & 0 \end{array}\right]$$

The Kalman update is computed with Equation (9), where **W** = **w** from Equation (A11) and **H** = **h**. From Equations (A7), (A11) and (A12), the Kalman update for the state vector equals the initial design-based estimate plus the weighted difference between the design-based estimate of the auxiliary variable and its exact census value from the enumeration in the GIS:

(A13) $${\left[\hat{x}\right]}_{KF}={\left[\begin{array}{c} {\hat{x}}_{1}\\ {\hat{x}}_{2}\\ \vdots \\ {\hat{x}}_{k-1}\\ {\hat{x}}_{k} \end{array}\right]}_{SRS}+\left({\left[\begin{array}{c} {\hat{V}}_{1,k}\\ {\hat{V}}_{2,k}\\ \vdots \\ {\hat{V}}_{k-1,k}\\ {\hat{V}}_{k,k} \end{array}\right]}_{SRS}\frac{1}{{\left[{\hat{V}}_{k,k}\right]}_{SRS}}\right)\left(y_{a}-{\left[{\hat{x}}_{k}\right]}_{SRS}\right)$$

where *y_a_* is computed with Equation (A3). The Kalman update for the state covariance matrix in Equation (11) is:

(A14) $${\left[\hat{V}\right]}_{KF}=\left[\begin{array}{ccccc} 1 & 0 & \cdots & 0 & -w_{1}\\ 0 & 1 & \ldots & 0 & -w_{2}\\ \vdots & \vdots & \ddots & \vdots & \vdots \\ 0 & 0 & \ldots & 1 & -w_{k-1}\\ 0 & 0 & \ldots & 0 & 0 \end{array}\right]{\left[\hat{V}\right]}_{SRS}\left[\begin{array}{ccccc} 1 & 0 & \cdots & 0 & 0\\ 0 & 1 & \ldots & 0 & 0\\ \vdots & \vdots & \ddots & \vdots & \vdots \\ 0 & 0 & \ldots & 1 & 0\\ -w_{1} & -w_{2} & \ldots & -w_{k-1} & 0 \end{array}\right]$$

Substitution of the weights in Equation (A11) into (A14) yields the algebraically equivalent form:

(A15) $${\left[\hat{V}\right]}_{KF}={\left[\begin{array}{ccccc} {\hat{V}}_{1,1}-\left(\frac{{\hat{V}}_{k,1}^{2}}{{\hat{V}}_{k,k}}\right) & {\hat{V}}_{2,1}-\left(\frac{{\hat{V}}_{k,2}{\hat{V}}_{k,1}}{{\hat{V}}_{k,k}}\right) & \cdots & {\hat{V}}_{k-1,1}-\left(\frac{{\hat{V}}_{k,k-1}{\hat{V}}_{k,1}}{{\hat{V}}_{k,k}}\right) & 0\\ {\hat{V}}_{2,1}-\left(\frac{{\hat{V}}_{k,2}{\hat{V}}_{k,1}}{{\hat{V}}_{k,k}}\right) & {\hat{V}}_{2,2}-\left(\frac{{\hat{V}}_{k,2}^{2}}{{\hat{V}}_{k,k}}\right) & \ldots & {\hat{V}}_{k-1,2}-\left(\frac{{\hat{V}}_{k,k-1}{\hat{V}}_{k,2}}{{\hat{V}}_{k,k}}\right) & 0\\ \vdots & \vdots & \ddots & \vdots & \vdots \\ {\hat{V}}_{k-1,1}-\left(\frac{{\hat{V}}_{k,k-1}{\hat{V}}_{k,1}}{{\hat{V}}_{k,k}}\right) & {\hat{V}}_{k-1,2}-\left(\frac{{\hat{V}}_{k,k-1}{\hat{V}}_{k,2}}{{\hat{V}}_{k,k}}\right) & \ldots & {\hat{V}}_{k-1,k-1}-\left(\frac{{\hat{V}}_{k,k-1}^{2}}{{\hat{V}}_{k,k}}\right) & 0\\ 0 & 0 & \ldots & 0 & 0 \end{array}\right]}_{SRS}$$

The Kalman estimate for the auxiliary variable ${\left[{\hat{x}}_{k}\right]}_{KF}$ in Equation (A13) equals the known census constant *y_a_*, and the *k*-th row and column of the covariance matrix after the Kalman update in Equation (A14) equals the zero vector. Therefore by definition, the updated “estimate” of the single auxiliary variable is orthogonal to the remaining study variables. In a heuristic sense, the Kalman update “filters” all information from the single auxiliary variable *k* that is linearly related to the remaining *k* − 1 state variables, which improves estimates for the study variables. Stated differently, the KF reduces the variance of population estimates for all state variables that are correlated with the auxiliary sensor variable.

### A.3. Constrained Kalman Weights for Divergent Outliers

The KF updates the estimated *k*-by-1 state vector with the weighted scalar difference between the known population values of the auxiliary variable *y_a_* and its current best estimate, as given in Equation (A13). The weights are optimal in the sense of the minimum variance estimator. However, the residual difference between its exact value, which is computed from the enumeration of all pixels and polygons for the sampled population with the GIS in Equation (7) and its sample survey estimate in Equation (5), can be unexpectedly large relative to its estimated variance. This would raise the suspicion that underlying assumptions do not conform well to reality; perhaps there is an unidentified source of substantial non-sampling error.

Consider the following example. Assume that the sampled target population can be accurately delineated within the Geographical Information System (GIS), which is necessary to enumerate the remotely sensed auxiliary data for each pixel and polygon in the sampled population. However, some sample plots are not measured because landowners deny access to the ground plot by the field crew. Other sample plots are not measured because they are permanently inaccessible due to field conditions, cliffs, slopes, *etc*. The NFI program cannot identify all lands that would hypothetically be inaccessible to a field crew had the landowners denied permission for an NFI field crew to access to their property. The target population can be accurately mapped, but not necessarily the sampled population. The mean remotely sensed characteristics of the target population might not exactly match those of the sampled population. This could produce a relatively large residual difference between a sample estimate of an auxiliary sensor variable and its true census value, which is computed with a GIS for the target population. Some unknown portion of that residual difference might be systematic non-sampling error.

We typically assume that the exact value for each auxiliary variable is known without error at the geographical location of each sample plot, which requires precise geographic coordinates for each ground plot. We further assume pixel data from earth-observing satellites and polygon data from other geographical sources are accurately registered to cartographic coordinates in the GIS. However, no georeferencing protocol is perfect, and systematic registration error is a potential source of unknown measurement error.

Therefore, contrary to the usual assumptions, some unknowable portion of the residual difference between the known population value of the auxiliary variable and its best current estimate could be caused by non-sampling errors. Engineering applications of the KF are likewise vulnerable to deviations from nominal assumptions. Engineers even coined a term for this aberration, “divergence.” Robust engineering applications use methods to detect and mitigate divergence.

Divergence causes too much weight to be placed on certain auxiliary variables because the realized sample survey estimate does not capture the consequences of non-sampling errors. Divergence is often caused by a variance for the estimate of an auxiliary remotely sensed variable that is nearly zero, which causes its inverse to become very large in Equation (A11). This can be common during sequential application of the Kalman update, which is explained in Section 4.

Divergence can also occur through random sampling errors. Here is an extreme example. Consider a remotely sensed characteristic that is rare. Pixels or polygons with this characteristic exist in the GIS, but they might not occur in the realized sample. In this case, the sample survey estimate for the prevalence of that attribute could be exactly zero with zero estimated variance, even though this rare characteristic truly exists in the target population. This is an example of a “sampling zero.” In less extreme cases, the sample survey estimate for a rare auxiliary sensor variable, and its estimated variance for random sampling errors, can be much smaller than their true values, which are knowable through enumeration with the GIS.

If the residual difference between the estimated and known population value of the *k*th auxiliary variable is large relative to its estimated variance, then divergence is likely. The following criterion is used to declare a residual as divergent based on the relative magnitude of the residual:

(A16) $$\left|y_{a}-{\hat{x}}_{k}\right|>\left(2{\left[{\hat{V}}_{k,k}\right]}^{0.5}\right)$$

In other words, if the absolute value of the residual difference exceeds two standard deviation units of its estimated variance, then assume the estimated variance is too small relative to its unknown expected value. Consequences of suspected divergence can be mitigated with a heuristic that scales the *k*th row and column of the state covariance matrix variance, like so:

(A17) $${\hat{V}}_{SRS}^{*}=\left[\begin{array}{cc} I & 0\\ 0 & \left(\frac{2\left|\;y_{a=k}-{\hat{x}}_{k}\right|}{{\hat{V}}_{k,k}^{0.5}}\right) \end{array}\right]{\hat{V}}_{SRS}{\left[\begin{array}{cc} I & 0\\ 0 & \left(\frac{2\left|\;y_{a=k}-{\hat{x}}_{k}\right|}{{\hat{V}}_{k,k}^{0.5}}\right) \end{array}\right]}^{\prime }\text{if }\left(\frac{2\left|\;y_{a=k}-{\hat{x}}_{k}\;\right|}{{\hat{V}}_{k,k}^{0.5}}\right)>1$$

The KF with this heuristic is suboptimal in the sense of a minimum variance estimator. However, the loss of efficiency is expected to be minor, and I believe this constraint is worth it because of its value as a mitigator for outlier estimates in the auxiliary variables. The algorithm in Section 4 includes this heuristic in lines 6 to 10.

### A.4. Chance Correlations

The algorithm in Section 4 is numerically stable with thousands of study variables and thousands of auxiliary sensor variables. This can reduce the variance of random estimation errors for any study variable that has a nonzero covariance with any auxiliary sensor variable. While this is the essential objective, this can also present problems.

For example, assume an auxiliary variable is strongly correlated with some study variables, but it truly has no correlation with other study variables. The true covariances between the latter and this auxiliary variable would be zero. Their estimated covariances will likely be near zero, but not exactly zero. Consider a remotely sensed indicator of disturbance at time *t = 2*. That auxiliary variable can be well correlated with study variables measured at time *t* = 2, but that same auxiliary variable could be uncorrelated with the same study variables measured at time *t* = 1.

Equation (A12) demonstrates that even a very small nonzero covariance will decrease the variance of an estimated study variable, although the decrease would be miniscule. However, what if there are numerous auxiliary variables that truly have no correlations with a certain study variable? Cumulative miniscule improvements to a study variable grow larger as more auxiliary variables are added. This can unintentionally reduce variance of study variables with auxiliary data that are truly uncorrelated but have an estimated nonzero empirical correlation.

To mitigate this phenomenon, certain elements in the covariance matrix estimated with Equation (6) can be modified. First, transform this covariance matrix into the conformable matrix of correlation coefficients. Next, conduct tests for the null hypothesis that each correlation coefficient equals zero [20]. If the null hypothesis that a correlation coefficient has the true value of zero is accepted, then replace the corresponding non-zero estimate of that covariance with exactly zero. The number of tests can be large: 2(*k_s_* × *k_a_*) + *k_a_*^2^, which means the algorithm will occasionally accept the null hypothesis when it is not true. However, there is no way to identify which null hypotheses suffer from these Type II inferential errors. A potentially small decrease in statistical efficiency might be necessary to reduce the risk from nonzero correlations caused by random sampling error. In the case study in Section 5, this null hypothesis (*t* = 1.96) was accepted for approximately 80% of the covariances between the *k_a_* auxiliary variables and the *k_s_* study variables.
